# Mechanisms and context in the San Patrignano drug recovery community, Italy: a qualitative study to inform transfer to Scotland

**DOI:** 10.1080/09687637.2020.1747397

**Published:** 2020-04-21

**Authors:** Alison M. Devlin, Daniel Wight

**Affiliations:** MRC/CSO Social and Public Health Sciences Unit, University of Glasgow, Scotland, UK

**Keywords:** San Patrignano, drug recovery community, mechanisms, complex intervention, transferability, context, programme theory

## Abstract

The San Patrignano drug recovery community, Italy, is regarded as one of the most successful in the world. However, if this model is to be transferred to other countries, it is necessary to clarify its underlying mechanisms and how far their success is context dependent. This qualitative study investigated these features of the San Patrignano model. Data collection included semi-structured interviews with six key stakeholders and 10 days’ observational field notes. Data were synthesised using frameworks and analysis was informed by realist principles. Individual level mechanisms include: commitment to change, removal from former social environment, communal living, peer mentor with lived experience and meaningful work. These operate in the context of a free of charge, long term (3–4 year) residential community. Organisational level mechanisms are: visionary leadership, staff dedication, social enterprise and adaptable learning. Organisational contextual factors include: a gap in suitable provision for drug recovery and the region’s high level of social capital. Articulating the programme theory of the recovery model and its contextual dependency helps clarify which elements should be transferred and how far they need to be adapted for different socio-cultural settings. The recognition of context is crucial when considering transfer of effective complex interventions across countries.

## Introduction

Drug addiction is a key social and public health challenge in Scotland with an estimated 58,900 problem drug users (Gao et al., 2016; NHS Scotland, Information Services Division (ISD), 2019; Parkinson et al., 2018). The incidence of drug related deaths has increased yearly in Scotland, which now has the highest rate in Europe, making this a public health emergency (National Records of Scotland (NRS), 2019). A harm reduction approach has been implemented for several decades, and plays an important role, but there is concern that methadone maintenance is contributing to drug related mortality (Gao et al., 2016; McCowan et al., 2009; Pierce et al., 2018). Problematic and extreme drug use is more prevalent in areas of socio-economic deprivation, related to structural stressors, such as unemployment, and a lack of other forms of social capital (Buchanan, 2004, 2006; Parkinson et al., 2018). There has been a shift in analysis of drug addiction from the deficit, disease-based model towards recovery-centred solutions that recognise the wider, social determinants of addiction (Alexander, 2000; Buchanan, 2006; Laudet & White, 2008). The concept of addiction recovery, its definition and ownership has been widely debated (Laudet, 2007; White, 2007). Despite re-alignment of government policy, with stronger emphasis on addiction recovery, defined by the Scottish Government as:

a process through which an individual is enabled to move on from their problem drug use, towards a drug-free life as an active and contributing member of society.

(Road to Recovery, Scottish Government 2008, 3, p23),

there is limited evidence of any real change in provision (Best et al., 2010; Kimber et al., 2010; Laudet & White, 2010). Previous research with drug addicts in Scotland highlighted their aspirations for drug free recovery (McKeganey et al., 2004), but there remains a lack of policy translation into practice due to competing service demands against a backdrop of wider austerity measures (McKeganey, 2014). Given the unprecedented drug-related mortality in Scotland, the need to find effective solutions is more urgent than ever, leading to interest in transferring successful, recovery-based models from other countries (Devlin & Wight, 2018).

Independence from Drugs and Alcohol Scotland (IFDAS) (http://www.ifdas.net/) is a charity founded to build a new model of recovery, based on social enterprise (Bitel, 2013). San Patrignano (https://www.sanpatrignano.com/) in Emilia Romagna, Italy, is one of the world’s largest and most successful drug recovery communities. A follow up study found 70% of those who completed the programme remained drug free after 4 years, measured using hair strand toxicological analysis (Castrignano, 2012; Manfre et al., 2005). San Patrignano was founded in 1978, by Vincenzo Muccioli; a social entrepreneur who identified a gap in provision for drug addicts whose marginalisation was exacerbated at that time by stigma and fear of HIV/AIDS (Perrini et al., 2010). San Patrignano has previously been referred to as a ‘third generation (European) therapeutic community’ (Broekaert et al., 2006). The San Patrignano model, which is based on social enterprise, now has approximately 1500 residents in recovery, and because of its success and longevity, has inspired IFDAS to establish a similar prototype community in Scotland (Bitel, 2013).

The therapeutic community (TC) originated in the United States as an effective psychosocial intervention for those with severe drug addiction problems (De Leon, 1995, 1996) and its evolution in Europe has been documented by Broekaert et al. (2006). The TC is based on ‘community as method’; a term used to describe the purposive use of community and its therapeutic processes based on mutual and self-help to effect change (De Leon, 2000). Although TC effectiveness has been documented by De Leon (2010), other studies have shown mixed results (Malivert et al., 2012; Sacks et al., 2003). A systematic review by Smith et al. (2006) showed limited TC effectiveness but there was marked heterogeneity across the included studies, in terms of programme length and treatment elements, as well as variation in target populations. In contrast, some comparative studies have demonstrated improved outcomes for the TC approach versus methadone maintenance (Babaie & Razeghi, 2013; Vidjak, 2003). A more recent review concluded that TCs are generally effective for substance use disorders, reducing substance use and criminal activity (Magor-Blatch et al., 2014). In keeping with a recovery-oriented perspective, Vanderplasschen et al. (2013) found some evidence of TC effectiveness for substance use and a range of outcomes across other life domains. However, one of the most consistent findings across the research field (also noted from San Patrignano) was the correlation between duration of time spent in a community and progression in recovery (De Leon, 2010; Manfre et al., 2005; Vanderplasschen et al., 2013).

Previous research studies on how San Patrignano works have focused on specific features related to *either* success of the organisation, *or* the therapeutic programme. For example, studies have investigated the economic model of social enterprise on which the organisation is based (Imperatori & Ruta, 2015; Perrini et al., 2010). Others have focused on specific therapeutic aspects, including a Foucauldian interpretation of ‘narrative therapy’ (Yates, 2014) and an interpretation of the residents’ immersion in community as a microcosm of wider society: the so-called ‘city effect’ (Guidicini & Pieretti, 1995). As far as we are aware, there have been no previous studies that attempt to synthesise these features in relation to their socio-cultural context, and at more than one level.

There is currently great interest in the transferability of social and public health interventions from one setting to another, especially those that address intractable complex issues such as drug addiction (Craig et al., 2018; Devlin & Wight, 2018). However, when considering the therapeutic community, De Leon has previously cautioned:

the global psychosocial ecology of the therapeutic community, the holistic nature of the disorder and the dynamic properties of recovery make describing, much less understanding, the TC process a formidable challenge (De Leon, 2000, pp. 105–106)

The realist evaluation approach conceptualises complex interventions as dynamic systems comprised of ‘mechanisms’ interacting with contextual features to produce outcomes (Pawson & Tilley, 1997). This is the first study to conceptualise the San Patrignano drug recovery model as a system, by drawing on realist principles to investigate mechanisms that contribute to its success at the individual and organisational levels, as well as the socio-cultural contextual features which support their function (Pawson, 2013; Pawson & Tilley, 1997). Accordingly, the aims of this study are to investigate the programme theory of the San Patrignano model in order to:

1. Identify the mechanisms that underpin the programme theory at the individual (micro) and organisational (meso) levels.
2. Investigate how these mechanisms interact with contextual factors at each level to contribute to positive outcomes.

Through doing so, this study will make a contribution towards a better understanding of how complex interventions work in relation to contextual features and which is key when considering their transfer. The study is part of a Medical Research Council (MRC) funded research programme on transferability of complex interventions between different contexts, and was conducted by researchers independent of San Patrignano or IFDAS.

## Methods

### Data collection

We conducted a qualitative research study which involved in-depth, semi-structured interviews with six key stakeholders at San Patrignano, sampled purposively for their extensive experience (from 11 to 38 years) of living and working in the community (Palinkas et al., 2015). As such, interviewees sampled were ‘information rich’ and with a cross-section of relevant knowledge and expertise in order to address the aims of the study (Malterud et al., 2016). It is important to highlight that the stakeholders are all in senior management/ leadership roles such as: CEO of a social co-operative; medical director of the San Patrignano hospital; head of international relations. Four of the stakeholders are ex-addicts and previous residents of San Patrignano, in long term recovery. Furthermore, three of the stakeholders are from the original founding group. Stakeholder expertise included: social enterprise, international drug policy, clinical expertise and therapeutic recovery methods. The interviews addressed features including stakeholders’ perceptions about the main mechanisms underlying the model (i.e. in your opinion, how do you think the San Patrignano model *works*?) and how these relate to context (Pawson & Tilley, 1997). In order to investigate wider contextual factors, interviewees were asked to comment on facilitators and barriers to the development and sustainability of the San Patrignano community over its 40 year history. Finally, stakeholders’ initial perceptions on transfer of the model were briefly explored. The first interview also piloted the interview schedule with only minor modifications required. Most interviewees (5/6) had reasonably good English, but one was assisted by an interpreter (a community resident nearing the end of their programme, fluent in English and Italian).

The semi-structured interviews were complemented by field observations conducted by AD during a continual 10 day immersion living and working inside San Patrignano, which generated 50 pages of field notes. Field observations started during an initial two day stay in the pre-admission centre; ‘Boticella di Novafeltria’ followed by continual immersion in the main San Patrignano community. Additional qualitative data included notes and related documents from a series of 12 lectures during the San Patrignano international workshop (March/April 2017); 10 of which were delivered by five of the interviewees. A seventh stakeholder (not interviewed) delivered two lectures. The lectures covered all aspects of the community including structural and functional features (e.g. pre-admission procedures, the role of sport and the arts) as well as the economic model of social enterprise on which San Patrignano is based. Interviews were digitally recorded, transcribed verbatim, and carefully proof read to ensure accuracy. Observational field notes and lecture notes were prepared as Word documents. All qualitative datasets were uploaded into QSR NVivo (Version 10) software for data management and analysis. Ethical approval was obtained from the Ethics Committee, College of Social Sciences, University of Glasgow. Fully informed, signed consent was obtained prior to face-to-face interviews.

### Data analysis

Data analysis involved a combination of both inductive and deductive approaches (Fereday & Muir-Cochrane, 2006; Miles & Huberman, 1994). This involved careful reading and re-reading interview transcripts and recording initial themes in analytical memos. Through iterative interrogation of the interview transcripts, we devised a coding structure that incorporated ‘units of meaning’, as grounded in the data, to form categories that were then structured to prior, higher order concepts of mechanisms, context and outcomes (Lincoln & Guba, 1985; Pawson & Tilley, 1997). Both AD and DW independently coded a sub-sample (2/6) of interview transcripts to ensure consistency of coding. Thereafter, comprehensive analysis was conducted, which involved coding all interview transcripts, observational field notes and lecture notes in QSR NVivo (Version 10). Regular meetings were held between authors to build a consensus understanding of findings. Data synthesis was guided by Framework Analysis distinguishing between findings at the individual (micro) and organisational (meso) levels and triangulation of data has been conducted wherever possible to enhance rigour (Patton, 2002; Ritchie & Spencer, 2002). The results draw on research interviews, observational field notes and lecture notes and are embedded within the results sections using anonymised descriptors in the following format: IKS_1–6: *Interview with Key Stakeholder (1–6);* OBSERV (Day 1-10): *Field observation notes (recorded on Day 1–10);* [KS_1–6]L1-12: *[Key Stakeholder’s_(1–6)] Lecture 1-12.*

**Figure 1:**
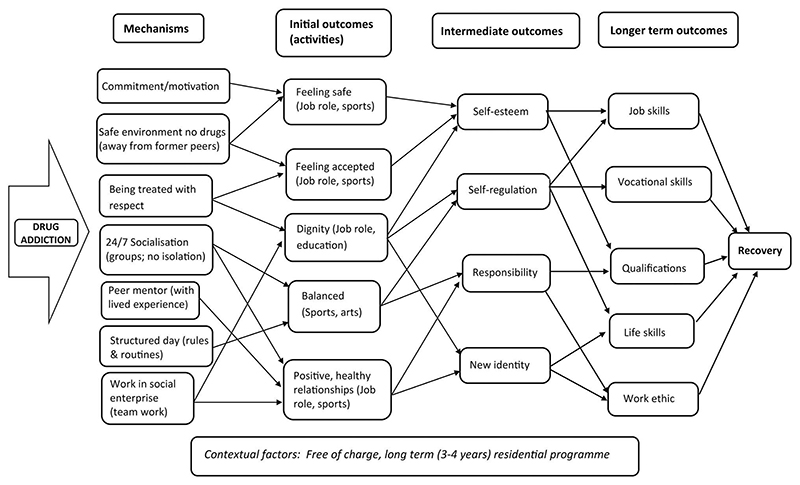
Model of the San Patrignano programme theory (individual or micro level).

**Figure 2:**
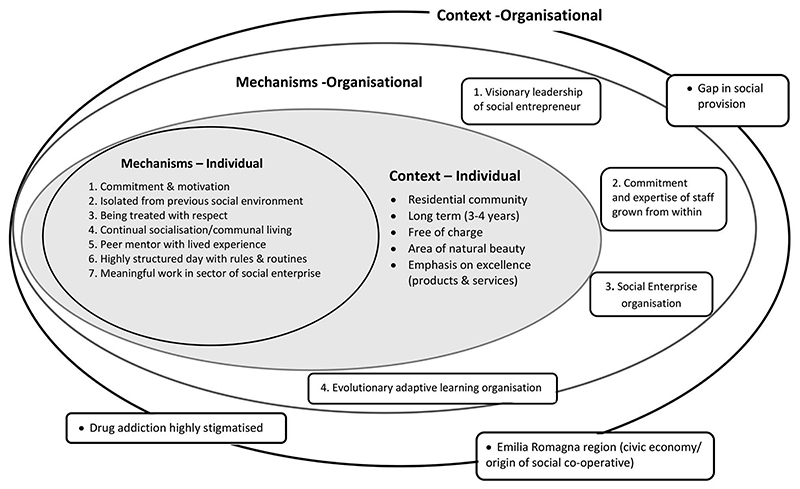
Schematic representation of the mechanisms and contextual features underpinning the San Patrignano model at the individual (micro) and organisational (meso) levels.

Drawing on the socio-ecological framework (Bronfenbrenner, 1977), we distinguish between mechanisms operating at the individual (micro) level, that contribute to the individuals’ recovery, and those at the organisational (meso) level, that are necessary for the San Patrignano community (as a whole) to be established and maintained. We summarise the key contextual factors within which these mechanisms work in Tables 1 and 2. We then constructed a model of the programme theory through mapping the links in the data between mechanisms and outcomes (Figure 1). This is a simplified conceptual working model of how the San Patrignano model is proposed to work (Rogers, 2008; Shearn et al., 2017). For clarity, the programme theory is presented in a linear manner however we acknowledge that in practice, it is a non-linear dynamic process with multiple interactions and feedback loops (Figure 1). Finally, we illustrate the complex nature of the San Patrignano model by presenting the mechanisms and context at the individual (micro) therapeutic level, as nested within the mechanisms and context at the organisational (meso) level (Figure 2).

## Results

Findings are presented as narrative and representative quotes and other data excerpts are included in each section. The three data sources revealed seven key mechanisms underlying the success of the San Patrignano programme at the individual level (Table 1).

### Individual level mechanisms (micro)

#### Commitment and motivation

Stakeholders (5/6) emphasised the importance of commitment to a new lifestyle free from drugs, with entry to San Patrignano being by self-referral and abstinence being an entry requirement, not an outcome. Although commitment is thoroughly assessed at the pre-admission stage, it is often unstable and has to be renewed frequently. Indeed, motivation often has to be renewed on a daily basis in the early stages and even over a number of months before it becomes intrinsic.

IKS_6: So when someone says ‘I want to go,’ we don’t say ‘Okay, go, please.’ No, we say ‘You don’t have to go, remember you decided to enter because of this and this.’ This choice has to be renewed continuously, because when confronting the difficulties, it is easy that you want to give up.

IKS_4: When I came here after the first months, it was very difficult for me because I had always thought I’m not able to, I cannot succeed in, so a lot of times I went out of this place, [San Patrignano] to take drugs. But after some time, I decided to stop. I don’t want to keep going out. I started believing in this way of life.

#### Removal from former environment

Interviewees highlighted the need to remove the individual from their existing environment and social networks to a new, safe residential environment that is drug-free. Residents are completely isolated from former peers; a radical change considered essential in order to begin the process of new identity formation and a key part of their recovery journey. New residents are given immediate, continual support within the San Patrignano community in a group of like-minded peers who are all in recovery, which fosters a sense of safety and belonging.

IKS_1: The therapeutic community requires a separation from the previous environment. In order to make the change start it is necessary to break with your previous attitudes and behaviour. In fact, the first theorist of therapeutic communities wrote it is necessary to have a total separation because you have to invest, to make the change start. Otherwise this change won’t start, never.

#### Being treated with respect/strengths-based approach

All stakeholders recognised the approach adopted in San Patrignano which is based on treating residents with respect and focuses on the potential of each person, and not on drugs. Initially, emphasis is placed on meeting the human being’s basic needs for food, safety and shelter which provides immediate relief from external structural stressors. In contrast to the disease-based deficit model, this holistic approach aims to re-build self-esteem in order to gradually foster agency in residents who are also encouraged to reflect on their future possibilities and take active ownership of their own recovery journey.

IKS_6: I think it works because it is tailored on the human beings needs. It was created […] to welcome those who needed support, who needed a new family, a home. The intention of the community is to welcome people and their needs, which are many. It’s not only getting rid of addiction. Addiction is just one.

[KS_4]L5: We tell them, it is your responsibility, your own desire that will make the change happen.

#### Continual socialisation/communal living

Field observation revealed continual socialisation in all community activities. Residents are organised in groups related to the sector (of the social enterprise) in which they work, and since they also share accommodation, they are never alone. Interviewees (5/6) agreed this is an essential part of the therapeutic approach since ongoing communication, sharing and interaction with recovery peers enables reflection and resolution through narrative processing, *‘like a gym for your mind, you have to … cope with crossing a lot of bad and good periods’* [IKS_4]. This also helps to support important social learning and formation of healthy, positive ‘family-like’ relationships.

IKS_5: We have a big dining room and we have lunch and dinner together because it’s like a family … and it’s important for a family to stay around a table, eating, and we stay together in San Patrignano when you work, when you are at lunch, when you have any free time, when you go to the music …

#### Peer mentor with lived experience

All interviewees referred to the crucial role of the peer mentor with lived experience who provides a credible role model with rational authority since the mentor is working on their own recovery journey, just further along the path. The dynamic of reciprocal respect promotes mutual understanding and provides an important opportunity for learning and development in both individuals. This was directly observed on a daily basis in both the pre-admission centre and in the main San Patrignano community.

IKS_4: I think it’s very important because people who come in the place (San Patrignano) believe in other people who have the same problems, because another one who had not been on the programme you could say you are different from him. But if you know a lot of people that have your same problem you cannot say it’s impossible.

OBSERV (Day 2): I saw one guy talking to a younger guy about cleaning the dining area, as if he was trying to convince him to join in. I then realised it was a mentor with new member. Eventually the new guy began to work along-side the others. It was calm and everyone seemed to know exactly what to do.

#### Highly structured day with rules and routines

Stakeholders (3/6) emphasised the importance of a highly structured day with rules and routines, in contrast to a typically unstructured drug-taking lifestyle in which: *‘addicts are not used to rules of any kind … they have to learn that you do not break the rules’* [KS_3]L3. Learning to undertake routine daily tasks reinforced over a long time period, helps build self-regulation and gaining a sense of reward from such activities. Interviewees also explained that tasks and responsibilities are gradually expanded as the individual demonstrates progress in recovery.

IKS_6: New pattern of behaviour, that has to become part of your life and influence your capacity of getting satisfaction from small things. For example, you have to clean your bedroom, every day and the first time you say ‘Oh, no, I don’t want to clean it, it’s not necessary!’ Then you see that a clean room gives you a good feeling […] and you learn that these kinds of things that you thought were not important are important actually, because maybe make you feel better …

Importantly, and as evidenced through field observation (Days 5 & 8), the rules and norms of behaviour are monitored and conflicts resolved by the residents, which fosters community ownership.

#### Work in a sector of the social enterprises

Interviewees (4/6) spoke about the importance of work as a pivotal mechanism in the recovery process. Team work in the sector of the social enterprise contributes to group cohesion, enhances responsibility and enables residents to build a strong work ethic. Residents make a valued contribution through production of high quality craft goods in a culture of excellence, which contributes to self-worth, as evidenced in field observation.

OBSERV (Day 5): I can see commitment, dedication, composure and pride in residents who are actively engaged in the sectors (bakery, graphics, stables, house furnishings, winery).

Social enterprise sectors are managed and monitored by residents themselves in an ethos of respect and inclusion. Finally, working in a sector of the social enterprise organisation is not only important in forming a new identity, but the acquisition of skills and an employment history also contributes to building recovery capital.

IKS_1: Because you have to help them get life skills, get job skills and help them get closer to the real world of work. Because otherwise they will impact negatively if they go outside the community and discover the real job market is different of what they have lived here. So […] you have to gradually prepare the guys before they go out to know what exactly work means. The ethic of work, the responsibility.

In addition to the mechanisms that contribute to how the San Patrignano programme works, interviewees (3/6) commented that the programme does not suit everyone, in particular individuals with serious psychiatric disorders. However, this is usually carefully assessed at the pre-admission stage, or determined in the pre-admission centre.

#### Context – individual – micro

For the individual, commitment to recovery from addiction starts in wider society outside San Patrignano, but it is reinforced and sustained in the community. The other six mechanisms detailed above are supported by three main contextual features of the San Patrignano model. First, it is a long term (3–4 year) residential programme, second it is non-linear and personalised since: *‘there are no pre-defined therapeutic steps because people are different from each other and need different timings’* [IKS_6] and third, it is completely free of charge to all residents.

IKS_5: It’s different because it’s a long term programme. In Italy we have a lot of facilities, mostly social services, but the programme is shorter than San Patrignano because the state has to pay for the people that are in the facility. For San Patrignano, no-one has to pay, not the families, not the people in the programme, or the state. So we can offer a long term programme.

These contextual factors are enhanced by San Patrignano’s beautiful rural location. The mechanisms and contextual features with which they interact are summarised in Table 1.

A conceptual model of the San Patrignano programme theory at the individual level is presented in Figure 1. This is a working model depicted in a linear manner for the sake of clarity. However, we acknowledge that in practice, this is a non-linear, dynamic process with multiple interactions and potential feedback loops.

#### Organisational level mechanisms (meso)

The three sources of data revealed four key mechanisms underlying the success of San Patrignano as an organisation (Table 2).

#### Visionary, entrepreneurial leadership

All interviewees agreed the founder, Vincenzo Muccioli was an inspirational leader and skilled social entrepreneur. In the context of the disease-based deficit model then prevalent, he identified a gap in social provision, took risks and was a catalyst for change. His approach was innovative since it centred on the person, not drugs and he advocated tirelessly against stigma and marginalisation. In keeping with his vision of community empowerment, he identified future leaders of San Patrignano amongst the first residents in the late 1970s. The founder also secured the support of wealthy patrons who provided significant financial resources.

IKS_6: He was very hard headed [stubborn]. He explained that he was right, because these people were human beings that needed help! When the first result started to be evident, that people became free of drugs, recovered, and one studied and become a doctor at the university, this helped [people] to see, maybe Vincenzo was right …

IKS_4: Another very important matter is that Vincenzo, I remember since the beginning, was looking for people like me […] that could be leader. He was trying to find a group of former drug user(s) that could be the right person in this place. He was always saying to me, ‘We need to find a group, because in the future, this group should be the leaders of San Patrignano.’

#### Commitment and dedication of staff

Interviewees (4/6) described the commitment and dedication which their role entails, with some describing it as a ‘choice of life’ or a ‘mission’ to help other people. Several referred to the need for humility and respect for new residents, reflecting that their own recovery is still ongoing: *‘my condition is not different from his condition’* [IKS_3]. This illustrates the organisational culture of mutual respect since most senior managers have come through the programme, have a deep understanding of the San Patrignano ethos, and adhere to it. However importantly, humility is balanced by a non-paternalistic approach by staff who continually emphasise personal growth and development.

IKS_4: The key factor was a group of friends, people that had the feeling to have a mission because, without this feeling, to open a place [like this] is not a job. It should be the most important thing that you want to do. It’s not because of religion, it’s not because of political idea, but the desire to help people. But also to understand that help is different to always say, ‘Yes’. Help could also be to say, ‘No, it’s not possible.’ So it is not, ‘Poor drug user, I want to help you!’ Not in this way, but in a stable way.

IKS_1: Humility, is fundamental because the latest that arrive in community, the newcomer, has something to teach you. Everyone can learn from each other. The idea that things can change […] always think that you can improve, and this is the same attitude you have to transmit to the guys, because the people in recovery maybe think, ‘Okay, I’ve reached this level, that’s enough.’ On the contrary, you have to continuously change and overcome your limits and when you stimulate them, at the same time you stimulate yourself.

#### Social enterprise

Stakeholders commented on the pivotal role of social enterprise which enables San Patrignano to be independent of government funding, allowing autonomy and flexibility to meet the residents’ needs compared to mainstream services.

IKS_6: Vincenzo didn’t want to have any sort of monthly fee […] contribution from the government, because this would link him to their rules. On the contrary, Vincenzo wanted to work according to this own vision .

The social enterprise organisation is on a large scale, generating approximately 50% of the organisation’s income and places great emphasis on excellent produce for external, high end markets. San Patrignano has a broad governance board committed to the challenge of balancing social impact with commercial activities. It is an organisation based on empowerment since leaders are, *′… trained inside San Pa to fulfil these tasks. By training inside they have learned that the skills and the values go together’* [KS_7]L8. San Patrignano has built many partnerships with expert external organisations and sponsors in each of the commercial sectors, but always strives to maintain social impact as its core mission.

IKS_1: We are working with the most important international chefs because they want to know our products, to use these products in their cooking. So it is a very important and useful relationship. But we never forget the main point is vocational training and life skills to be given to the residents of the community. This is the core mission and … all that has [been] added has been to help this mission be fulfilled in a better way.

#### Evolutionary, adaptive learning

All stakeholders emphasised that the successful development and sustainability of San Patrignano depended on continuous experiential learning and adaptation. They recalled its organic evolution and ability to learn from its mistakes over its 40 year history.

IKS_5: We have 40 years of experience and we built, little by little, day by day. But we made a lot of mistakes, even with the economics, with resources . You have to start and you have to learn from your mistakes.

The community has adapted to meet residents’ needs such as changing type(s) of addiction (heroin, cocaine, poly-drug use) as well as the changing needs for new commercial sponsorships essential for financial sustainability. Interviewees also reported that residents were involved in establishing community rules over the years, which were either revised or reinforced in terms of what was found to work in practice.

IKS_3: Step by step, year to year, the people learned […] learned the problem. Because in 1980 the big problem was heroin and then it changed [to] cocaine, and then it changed [to] chemical drugs, and then it changed etc. So, its been a learning process … how to do, what to do, how to behave … also, to decide which rules to put …

#### Context –organisational – meso

The mechanisms involved in San Patrignano’s success at an organisational level are located within a wider historical and socio-cultural context (Table 2). In particular, the community is located in the Emilia Romagna region which has a strong tradition of civic economy and is where the Italian social co-operative movement originated. The region has a strong tradition of agriculture and skill in artisan produce as economic activity.

IKS_2: This area in particular, it’s probably one of the most developed in terms of economic exploitation of agricultural activity and there is a high level of social capital in this area. This is why, as [name of key stakeholder] said the movement of social co-operatives was born in this area.

San Patrignano was founded at the height of the ‘war on drugs’ when recovery oriented provision was unorthodox and rare. The newly developing community was treated with fear and suspicion, since addicts were highly stigmatised at that time. However, San Patrignano’s success and its leadership’s continual advocacy at the policy level for more recovery-based solutions, has led to its recognition by other services (including social services, the police and other government bodies) which now work in partnership with San Patrignano.

IKS_3: For example, the people of [name of city] and surrounding area, at the end of the seventies, a community full of drug addicts in our region … they were scared! Lots of difficulties created from fear, from not understanding and […] it was the years of heroin. So, it was a big problem. But with time, in Italy, the government have actually come close. When they understand then they support … and when they’ve got to know the importance of a place like this… how many people have been through the course, now we have the support of the police, the law, the government, social workers.

The way in which the four key mechanisms at an organisational level interact with contextual factors are summarised in Table 2.

The main mechanisms and the aspects of context at *micro* and *meso* levels are depicted schematically in Figure 2. We have drawn on an ecological perspective to illustrate how San Patrignano operates overall as a complex intervention (Westhorp, 2012).

#### Transferability of mechanisms to the Scottish context

We explored stakeholders’ perceptions about mechanisms that could be transferred to the Scottish context. Most stakeholders (4/6) agreed that the main elements including: the safe community free from drugs, and the strengths-based approach which treats people with respect should be transferred. They also commented on the importance of the recovery peer mentor with lived experience. Although interviewees confirmed the important function of work in the social enterprises and other daily activities in San Patrignano, they emphasised the exact form of these mechanisms would need to be adapted to suit the Scottish context.

IKS_4: Okay. I was thinking about this possible question some days ago … So I think that you should create a safe environment and the safe environment I think could be the same anywhere. But, em, you should choose the actual activities and the way of rewards that could be different from a place to another place.

One stakeholder expressed some concern about continual social interaction for Scottish people in recovery: *‘Maybe there is not this grande familia way of staying together’* [IKS_6]. Related to this, two stakeholders mentioned the wine served at meals in San Patrignano as being an important aspect of Italian culture, but they had limited knowledge of the negative outcomes of alcohol use in Scotland.

IKS_5: We drink wine at lunch and dinner, but we don’t drink wine because we want to get drunk. We drink alcohol because it’s a pleasure […] and it’s a part of Italian culture. It’s important.

Two interviewees referred to ‘Basta’ (https://english.basta.se/); a drug recovery community in Sweden that was also inspired by San Patrignano but adapted to suit the Swedish cultural context, including their distinctive welfare system. Stakeholders mentioned the crucial importance of an inspirational, driving leader with entrepreneurial skills like Vincenzo Muccioli in establishing the community. Finally, most interviewees (4/6) emphasised the importance of evolutionary, adaptive learning as an essential mechanism to transfer and the need to: *′… find your own way … your Scottish way’* [IKS_5].

## Discussion

In this study, we present a model of the programme theory underlying the San Patrignano drug recovery community, Italy. As far as we are aware, this is the first study to conceptualise the model as a system, *drawing on* realist principles to address mechanisms and their interaction with contextual features in order to capture its complex nature (Hawe, 2015; Pawson, 2013; Pawson & Tilley, 1997; Rutter et al., 2017). On the basis of key stakeholders’ perceptions and other data, we have built a model programme theory (Figure 1) which consists of seven mechanisms at the individual level: (1) commitment to recovery; (2) removal from previous drug-taking environment; (3) being treated with respect; (4) continual socialisation/communal living; (5) recovery peer mentor with lived experience; (6) structured daily routine and (7) meaningful work in social enterprise. Results indicated four key mechanisms at the organisational level: (1) visionary leader with entrepreneurial skills; (2) staff commitment and dedication; (3) social enterprise and (4) evolutionary adaptive learning. Each of these mechanisms interacts with contextual features as summarised in Tables 1 and 2. Through investigating several mechanisms at the individual (micro) and organisational (meso) levels, this study then synthesizes them with contextual features to illustrate how San Patrignano operates overall as a complex intervention (Figure 2).

The mechanisms summarised above help in the formation of increased agency and progress towards recovery through a process described by De Leon (2000) as *‘assimilation, immersion and emergence*’. Formulation of a new identity begins immediately upon admission when emphasis is placed on addressing the human being’s basic needs (e.g. for food, safety, shelter) as opposed to their prior drug-taking identity (Biernacki, 1986; Maslow, 1970). Through collective group working and routines, residents adopt new behavioural norms and develop self-regulation through social learning and social control with continual reinforcement by likeminded peers (Moos, 2007, 2011). This helps build new healthy relationships and construction of a socially mediated recovery identity (Best, Beckwith, Haslam, et al., 2016a; Yates, 2014). The synthesis of cognitive, social and psychological learning required during the recovery process takes time (Bandura, 1971; Moos, 2007, 2011). The San Patrignano programme is long-term, (3–4 years) with no pre-defined therapeutic steps, since it is personalised and often messy and non-linear. This is in keeping with findings that refer to the recovery journey as a dynamic personal process (Best et al., 2011; Laudet & White, 2010; Rettie et al., 2020). Motivation and commitment to a recovery lifestyle is reinforced by likeminded peers, and other activities, such as meaningful work in the social enterprises and sports, contribute to building recovery capital (Cloud & Granfield, 2008). Although recovery mechanisms for the individual such as, peer support, work and meaningful activities have been reported previously, they were largely addressed as separate processes or in combination with a few others (Best et al., 2008, 2012, 2013; Laudet & White, 2008). In contrast, this study is the first to synthesise recovery mechanisms that operate at different levels (individual and organisational) with their socio-cultural contextual factors. For example, there have been previous accounts of organisational mechanisms, such as social enterprise initiatives (Best, Beswick, Hodgkins, et al., 2016b) but no conceptual analysis of how social enterprise serves as a therapeutic mechanism for the individual, as well as an effective economic mechanism, crucial to the sustainability of the organisation over a 40 year period. This analysis illustrates the interdependency of mechanisms and context which contributes to the San Patrignano community functioning effectively overall (Figure 2).

San Patrignano is a complex intervention that consists of multiple interacting components and, as such, cannot be overly codified (De Leon, 2000; Guidicini & Pieretti, 1995). Nevertheless, in order to transfer it to Scotland (or elsewhere), mechanisms identified by stakeholders such as removal from drug using networks, recovery peer mentors, meaningful work and social enterprise were thought essential, even if some need to be adapted. As Hawe (2015) has previously argued, as long as the function of key mechanisms is transferred, their form can be adapted (Hawe et al., 2004). Thus, if their function is not impaired, the specific form of mechanisms, such as meaningful work or exact types of sports, can be adapted to suit the new context. Stakeholders from San Patrignano also recommended that the vision of respect and the non-paternalistic ethos of the recovery approach is essential in order to foster agency in residents, community empowerment and growth from within (Zimmerman, 2000).

It is important to consider some limitations to the present study. First, all interviews were conducted and other data collected by AD, who did not speak Italian. Most stakeholders (5/6) had reasonably good English and an interpreter (a resident fluent in English and Italian) helped in one of the interviews. However, the researcher could not ascertain whether the presence of the interpreter had influenced the stakeholder’s responses in this one interview. This also posed a limitation in fully understanding residents’ interactions during the field observations. Second, the sample of stakeholders interviewed was small, and did not include any who may have had a less successful experience with the San Patrignano programme. Thus, there is a risk of bias in the sample and we did not interview any current residents to explore their perceptions of key mechanisms. Resources and time constraints precluded this. Therefore, this study should be considered as exploratory with the programme theory developed as a working model in order to inform transfer. Similarly, although we cannot comment on differential effectiveness of the mechanisms identified (since the present study was designed to investigate *how* the model works), a current outcomes study of the San Patrignano programme reports high retention with 26.2% drop-out and suggests this occurs in relatively older residents who have young children (Explora Research, 2018, pp. 11, 26). This high retention rate is in keeping with previous studies on San Patrignano (Castrignano, 2012; Manfre et al., 2005). Therefore, while our study has focused on ‘*what* works’, it has not investigated ‘for *whom’* it works (Pawson & Tilley, 1997).

However, these limitations must be balanced by some key strengths of this study. First, this independent study is one of the first to investigate mechanisms at both micro and meso levels in order to form a holistic conceptual model of *how* San Patrignano works. Second, although the sample size was small, the interviewees were sampled purposively, on the basis of their extensive experience of living and working in San Patrignano, and may be considered ‘information rich’ in order to address the research aims of the current study (Malterud et al., 2016; Palinkas et al., 2015). Indeed, four stakeholders in senior management roles were themselves ex-addicts in long term recovery and had come through the San Patrignano programme. Three stakeholders interviewed were from the original founding group, thereby capturing their perceptions of crucial historical and socio-cultural knowledge about the programme. Third, although interviews were not conducted with current residents, this was compensated by AD carefully observing and recording notes on community life, while living and working along-side residents (some of whom spoke English) over a continual ten day period. Finally, notes were also recorded during a series of 12 lectures delivered (in English) on all aspects of community life and work by stakeholders as part of the international workshop (March/April 2017). Therefore in keeping with realist evaluation principles (Wong et al., 2016) we drew on several forms of qualitative data, framed the analysis according to ‘mechanisms’ and ‘context’ and triangulated our findings across the three data sources to enhance rigour (Patton, 2002; Pawson & Tilley, 1997). The present study illustrates how the San Patrignano model relates to contextual features, which is critical in understanding how the intervention works.

There is currently great interest in the transferability of complex interventions, and in particular, the role of context (Craig et al., 2018; Devlin & Wight, 2018; Pfadenhauer et al., 2017; Schloemer & Schroder-Back, 2018). Articulating the programme theory underlying the San Patrignano model in terms of mechanisms and context is the first step in investigating the transfer of this intervention and whether the adaptations necessary can be identified in advance. We are aware that this is counter to the therapeutic community research culture which resists overt codification (De Leon, 2000) and we concur with a non-linear systems approach (Hawe et al., 2004; Rutter et al., 2017). Sniehotta et al. (2017) have previously cautioned against the false dichotomy of individual versus systems level interventions to public health challenges, rather emphasising their interdependent synergism to effect change. Such an approach also aligns with the most recent studies on addiction recovery (Best & Colman, 2019; Dekkers et al., 2019). The mechanism of social enterprise transcends dichotomy in the San Patrignano model, illustrating its nature as a complex intervention that involves fluidity between the individual (agency) and organisational (structural) levels to effect and support recovery (Figure 2). Indeed, the scale of the social enterprise that underpins San Patrignano is notable when compared with other TCs for addiction in Europe (EMCDDA, 2014) or globally (Imperatori & Ruta, 2015) and enables the programme to be long term and free of charge. In this study, we have attempted to explicate the tacit expertise that is embedded and enacted in daily practice in order to contribute to the research evidence base in the field. Furthermore, it is interesting to note that San Patrignano provides an example of a community empowerment-based recovery model which is mainly led by ex-addicts in long term recovery and is based on social enterprise which has enabled it to stay true to its vision. As such, there are parallels with a contemporary conceptualisation of the recovery movement as a form of pre-figurative politics (Beckwith et al., 2016) in which grassroots recovery groups take collective action to address their unmet needs and demonstrate viable, meaningful alternatives in order to influence change.

## Conclusions

This analysis of the programme theory of the San Patrignano recovery community shows that key mechanisms can be understood as operating at two levels: the individual and the organisational. At each level, the mechanisms operate in relation to different contextual factors and in some cases, may be a response to those contextual factors. Through clarifying the mechanisms of this recovery community and examining how their operation is context-dependent, this study will assist attempts to transfer the model to other countries. In particular, our next step will be to ascertain whether key stakeholders from IFDAS (http://www.ifdas.net/) can prospectively identify mechanisms considered essential for a recovery model being developed in Scotland, and how far differences in context will require them to be adapted (Devlin & Wight, 2018). We hope this work will contribute to policy for long term solutions using a strengths-based, empowerment approach (*Rights, Respect and Recovery*, Scottish Government, 2018) with the ultimate aim of improving the quality of life for all of those in recovery. More broadly, it is hoped this case study will contribute to the development of generic principles on the transferability of complex interventions across countries (Craig et al., 2018). This is of key importance as policy makers, practitioners and NGOs increasingly look to find successful models in other countries as possible solutions in their own setting.

## Figures and Tables

**Table 1:** Mechanisms and contextual factors: individual level.

Mechanism	Contextual factors
1. Commitment and motivation to recovery	Closed residential community Long term (3– 4 years) nature of programme Free of charge Community environment of like-minded peers
2. Removal from former social environment	Closed residential community Isolated, beautiful rural location Community environment of like-minded peers
3. Being treated with respect	Strengths based approach focuses on potential of human being Ethos of care, mutual respect No stigma, all residents in recovery
4. Continual socialisation/ communal living/ groups	Shared accommodation with little privacy Continual social interaction (e.g. working, eating, recreation) Narrative therapy in community of recovery peers
5. Peer mentor with lived experience	Self-regulating social community
6. Highly structured day with rules and routines	Large scale means whole community shares a highly structured day Community organised with precision and run very professionally
7. Meaningful work in sector of social enterprise	Over 50 work sectors in social enterprises producing high quality goods and services Ethos of craft excellence conducted within professional work environment reinforces value and self-worth Self-regulating working community Rural location and element of working on the land

**Table 2:** Mechanisms and contextual factors: organisational level.

Mechanism	Contextual factors
1. Visionary, entrepreneurial leader with wealthy patrons	Dominant, disease-based deficit model Gap in provision Recovery oriented provision unorthodox and rare Mistrust and suspicion
2. Commitment and dedication of staff	Senior managers largely recruited from residents Almost all senior managers are former addicts in recovery
3. Social enterprise	Region in Italy where social co-operatives originated History of excellence in agricultural and artisan produce Region with high level of social capital
4. Evolutionary adaptive learning	Independent from government funding, so can more readily adapt to suit the needs of the residents.
